# Prognostic impact of minimal extrathyroidal extension in papillary thyroid carcinoma

**DOI:** 10.1097/MD.0000000000005794

**Published:** 2016-12-30

**Authors:** De-Tao Yin, Kun Yu, Run-Qing Lu, Xianghua Li, Jianhui Xu, Mengyuan Lei

**Affiliations:** a Department of Thyroid Surgery, the First Affiliated Hospital of Zhengzhou University; b Key Discipline Laboratory of Clinical Medicine Henan; c Department of Hematology, the First Affiliated Hospital of Zhengzhou University, Zhengzhou, PR China.

**Keywords:** extrathyroidal extension, meta-analysis, papillary thyroid carcinoma, recurrence

## Abstract

**Background:**

It is widely accepted that maximal extrathyroidal extension (ETE) plays a vital role in the prognosis of papillary thyroid carcinoma (PTC). However, there is no consensus among researchers about the meaning of minimal ETE (mETE) in PTC. Herein, we conducted a systematic review and meta-analysis to examine the role of mETE in the prognosis of PTC.

**Methods:**

We searched PubMed, EMBASE, and Cochrane search trials databases in English to **identify** studies comparing data on disease recurrence in PTC patients with mETE and those with no ETE. To summarize the data related to mETE status, **risk ratios and hazard ratios adjusted for potential confounders were used to assess the number of recurrence and time-dependent risks related to mETE status, respectively**.

**Results:**

According to the inclusion criteria, a total of 7951 patients from 9 studies were included. The recurrence rate in patients with mETE is significantly higher when compared with those with no ETE (risk ratio = 1.70, 95% confidence interval: 1.26–2.28, I^2^ = 56%). **According to the data summarized with hazard ratios**, PTC patients with mETE **showed** a significantly increased risk of disease recurrence.

**Conclusion:**

**mETE is a risk factor for poor prognosis in patients with PTC.** Our innovative classification of ETE has its value in assessing the prognosis of PTC.

## Abstract 2

Supplemental Digital Content is available in the text

## 1 Introduction

As the most common thyroid malignancy, **papillary thyroid carcinoma** (PTC) is known to have a favorable prognosis with a cancer-related mortality rate <10%.^[1]^ For the past few years, the numbers of patients suffering from thyroid cancers have increased significantly.^[2]^ In 1961, Woolner et al first drew attention to the unfavorable prognosis of patients with PTC, whose “locally and highly infiltrative” tumors showed evidence of extrathyroidal extension (ETE).^[3]^ In 1986, McConahey et al reported that patients with PTC who were discovered to have maximal ETE at surgery were at an increased risk of recurrence and mortality, and those with maximal ETE had a “25 times greater chance of dying of PTC” than those with surgically intrathyroid tumors or a worse outcome (29% vs 9% disease recurrence) when all patients with ETE received adjuvant postoperation **radioactive iodine** therapy.^[4]^

Since then, it has been widely accepted that **patients with PTC who were discovered to have maximal ETE by a surgeon in surgery** have an increased risk of tumor recurrence and death from PTC.^[5–7]^ ETE has been accepted to be an important factor influencing the prognosis of thyroid cancers. Extensions to thyroid capsule, perithyroidal soft tissue, or sternothyroid muscle are classified as **minimal ETE** (mETE). Extensions to trachea, larynx, esophagus, recurrent laryngeal nerve, or subcutaneous soft tissue are classified as maximal ETE according to the seventh edition of tumor, node, metastasis system classification by the American Joint Committee on Cancer. **PTC with** mETE is upgraded to T3, and **PTC with** maximal ETE is classified to T4a.^[8]^ Although some researchers have reported that mETE is related to an increased rate of **recurrence and recurrence-free survival or relapse-free survival (RFS)** in PTC,^[9–11]^ other authors have observed that there is no significant difference in the disease-free survival (DFS) between patients with mETE **or** maximal ETE.^[12,13]^ Therefore, clinicians are unclear **about** the role of adjuvant therapy in light of the mETE in PTC, such as what should they do if intraoperative mETE is detected: should they take more radical measures or adopt a conservative strategy?

To evaluate the impact of the mETE on the prognosis of PTC, a systematic review and meta-analysis was conducted, in terms of disease recurrence and RFS or DFS. It was hypothesized that patients with mETE suffered from **worse** prognosis than those without it.

## 2 Materials and methods

### 2.1 Data sources and literature search strategy

Two investigators (KY and R-QL) searched PubMed, EMBASE, and **Cochrane search trials** (CENTRAL) in English independently. **Prospective or retrospective studies that compared indices of prognosis (disease recurrence, including malignancy recurrence confirmed by pathology, metastasis in other parts discovered by imageology during follow-up) in PTC patients with mETE versus those without ETE from the inception of the databases to June 21, 2016, were identified.** We searched the controlled vocabulary terms and keywords: (“extrathyroid” OR “extrathyroidal” OR “extracapsular”) AND (“Papillary thyroid carcinoma” OR “Papillary Carcinoma of Thyroid” OR “Thyroid cancer, papillary” [Supplementary Concept]) in PubMed, and in EMBASE and CENTRAL similarly. To include all qualified articles, references of the articles identified and conference abstracts were also examined and the related materials were **manually** searched. We have registered this study in the International Prospective Register of Systematic Reviews, and the registration number is CRD42015030149. All analyses were based on previously published studies; thus, no ethical approval and patient consent **were** required.

### 2.2 Study selection

In this meta-analysis, inclusion criteria were as follows: prospective or retrospective, observational cohort studies, comparing prognostic factors between mETE and no ETE (ETE was graded to the following: mETE that extended to the perithyroid soft tissue sternothyroid muscle, or the thyroid capsule, and maximal ETE that extended to the larynx, esophagus, mediastinal vessels, subcutaneous soft tissue, trachea, recurrent laryngeal nerve, prevertebral fascia, or carotid arteries^[8]^), diagnosis of PTC, and data on disease recurrence and RFS or DFS. Exclusion criteria included the following: no PTC, lack of prognostic parameters in the title/abstract, in vitro or animal studies, no data between mETE and those without it, studies with only maximal ETE or no ETE, and papillary microcarcinoma with a diameter of 1.0 cm or less.

### 2.3 Data extraction

Key data were extracted from the articles identified by 2 authors (KY and R-QL) independently. For each article, we extracted information as follows: the year of publication and the first author's name, the study population and nationality of PTC patients, the detection method and maximum follow-up time, and **hazard ratios** (HRs) associated with mETE for RFS/DFS. To extrapolate HRs with 95% confidence intervals (CIs), **data were extracted from graphical survival plots when only Kaplan–Meier curves were available**, with the method described by Tierney et al,^[14,15]^**in which necessary data were extracted** from the Kaplan–Meier curves with the free software, Engauge Digitizer, version 4.1 (free software downloaded from http://sourceforge.net), **and** the data of log(HR) and standard error in the above-mentioned way. **The extracted data were entered** into a standardized Excel (Microsoft Corp) file and examined by another author (D-TY). Disagreements were resolved with discussion and consensus. All of the data mentioned are listed in Table 1.

**Table 1:**
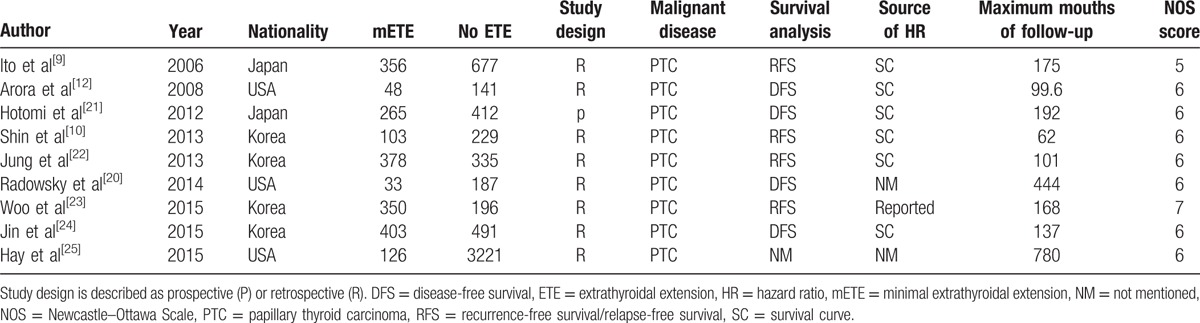
Main characteristics of studies included in the meta-analysis.

### 2.4 Outcomes

The primary outcomes were **risk ratios** (RRs) for recurrence of mETE versus no ETE. Secondary outcomes were HR for recurrence, adjusted for DFS/RFS rate in patients with mETE versus patients with no ETE.

### 2.5 Study quality assessment

To evaluate study quality, the Newcastle–Ottawa Scale^[16]^ was adopted, in which an increased risk of bias was denoted by a score of ≤5 (out of 9) (Table 1). This meta-analysis was strictly performed according to the Preferred Reporting Items for Systematic Reviews and Meta-Analyses statement.^[17]^ Any discrepancies were resolved by a consensus reviewer (D-TY).

### 2.6 Data synthesis and statistical analysis

Analysis was performed with Review Manager V5.3.5 (The Cochrane Collaboration, London, UK). Pooled RRs and 95% CIs for disease recurrence between mETE and patients with no ETE were calculated in primary analysis. To offer any necessary information in case that the relation between mETE and prognosis was affected by possible confounders, pooled HR with 95% CIs adjusted for the maximum number of covariates accessible in the articles was calculated in the secondary analysis. According to the condition of heterogeneity, Mantel–Haenszel method and the fixed-effects model or random-effects model were adopted.^[18]^ The heterogeneity test was verified with the Cochran Q test and it was quantified with the Higgins I^2^ statistic. When significant heterogeneity existed (*P* < 0.10 or I^2^ > 50%), a random-effects model was applied; in other cases, the fixed-effects model was utilized.^[19]^ Two-sided test was used to calculate *P* values, **and** it was statistically significant when the *P* value **was** <0.05.

## 3 Results

### 3.1 Search results

**Figure 1:**
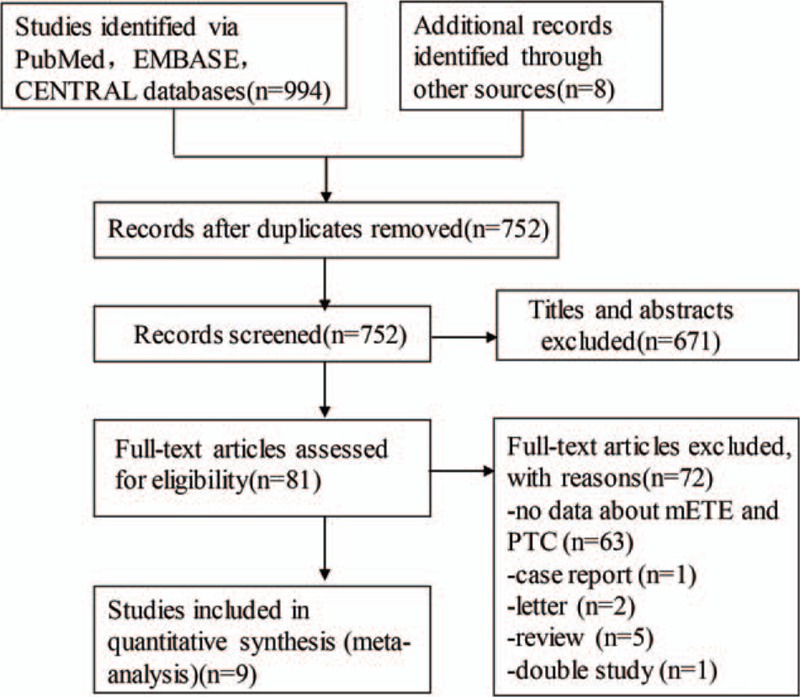
PRISMA flow diagram of study selection process. mETE = minimal extrathyroidal extension, PRISMA = Preferred Reporting Items for Systematic Reviews and Meta-Analyses, PTC = papillary thyroid carcinoma.

The search strategy described earlier yielded 944 abstracts from the PubMed, EMBASE, and CENTRAL database search and 8 from conference abstract booklets and reference lists in total. After exclusion of 250 duplicate reports, 752 abstracts were reviewed. Of these, 81 articles were reselected and the full articles were reviewed. And 9 were identified to be eligible for the meta-analysis. Figure 1**shows** the Preferred Reporting Items for Systematic Reviews and Meta-Analyses flowchart.

### 3.2 Study and patient characteristics

In total, 9 studies^[9,10,12,20–25]^**concerning** 7951 patients (2062 mETE and 5889 no ETE) were included in this meta-analysis. The authors of the included studies were from the USA^[12,20,25]^ (n = 3), Korea^[10,22–24]^ (n = 4), and Japan^[9,21]^ (n = 2). Of the 9 included studies, 1 directly reported HRs, **6 required estimation from survival curves, and** another 2 studies **had** no data about RFS or DFS curves. During this process, data were segregated according to either RFS or DFS. **Eight**^[9,10,12,20,22–25]^**of them** were retrospective cohort studies and **1**^[21]^ was prospective cohort study, and all were from hospital clinics. None were population, cancer registry, or pathology archive based. The average quality assessment score was 6 points, and 1 study was at a potentially high risk of bias (Table 1). However, **there was no randomized controlled trial** and most were retrospective cohort studies. **Therefore**, there might be an increased risk of bias in the studies. To reduce the heterogeneity of methodology and increase the reliability of the study, we analyzed the data from the retrospective studies in Section 2.6, and made a descriptive analysis of the prospective study in Section 4.

### 3.3 Disease recurrence

**Figure 2:**
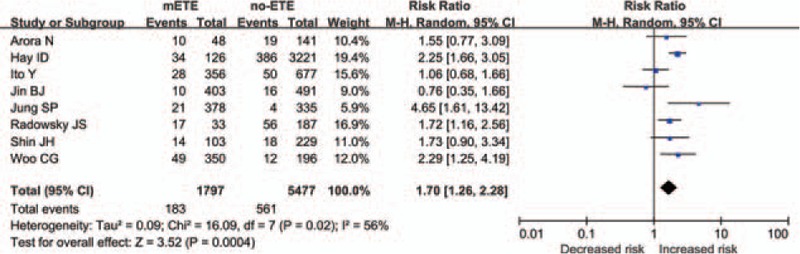
Forest plot RR of recurrence in association with mETE in PTC. CI = confidence interval, df = degree of freedom, mETE = minimal extrathyroidal extension, PTC = papillary thyroid carcinoma, RR = risk ratio.

Pooling data from the 8 included studies,^[9,10,12,20,22–25]^ the recurrence rate of patients with mETE was 10.18%, **lower than 10.24% of those without it**, indicating a significantly higher disease recurrence risk for the former (Fig. 2; RR = 1.70, 95% CI: 1.26–2.28, *P* = 0.004, I^2^ = 56%). **The risk difference was also evaluated** (supplemental figure; risk difference = 0.06, 95% CI: 0.01–0.10, *P* = 0.008, I^2^ = 83%), **which were supportive of our analysis**.

### 3.4 Adjusted HR for disease recurrence

**Figure 3:**
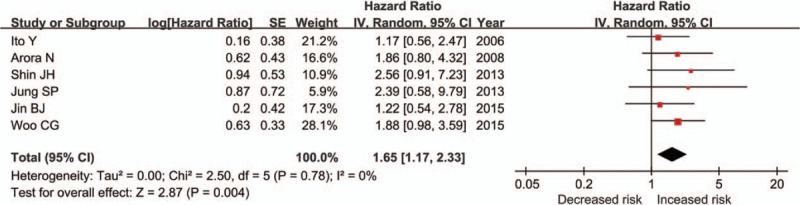
Forest plots of analyses with adjusted HRs of RFS or DFS in association with mETE in PTC. CI = confidence interval, df = degree of freedom, DFS = disease-free survival, HR = hazard ratio, mETE = minimal extrathyroidal extension, PTC = papillary thyroid carcinoma, RFS = recurrence-free survival or relapse-free survival, SE = standard error.

**It was also investigated whether our result would** be affected when the adjusted HRs were used instead of RR. The result showed that mETE was related to an increased risk of worse DFS in patients with PTC after initial surgery in 7 studies,^[9,10,12,22–24]^ leading to a significantly unfavorable prognosis (Fig. 3; HR = 1.65, 95% CI: 1.17–2.33, *P* = 0.004, I^2^ = 0%).

### 3.5 Sensitivity analyses

Sensitivity analysis was performed to examine the influence of individual studies. To assess the robustness of the results of this review, the meta-analysis estimates were derived by omitting 1 study each time. This led to exclusion of 1 study with suspected reporting bias.

## 4 Discussion

ETE, which is observed in 5% to 45% of PTC patients,^[26]^ has been reported to be an important factor related to poor prognosis.^[27–29]^ Now, it is known that ETE is divided into 2 grades: mETE and maximal ETE. Some researchers have reported that maximal ETE decreased the DFS of patients with PTC, while mETE has no impact on it.^[9,30]^ However, there are studies suggesting that patients with mETE had an increased risk of tumor recurrence.^[31,32]^ Therefore, it remains controversial whether more radical measures should be taken among patients with mETE than among those without ETE.

To our knowledge, our study is the first systematic review and meta-analysis to investigate the poor prognostic impacts of mETE in PTC. Nine observational **studies** were included, in which 1797 cases with mETE and 5477 without ETE were analyzed. In each of these studies, macroscopically complete tumor removal was performed. To evaluate intrathyroidal spread of PTC, ultrasonographical examination was performed routinely before operation. If the tumor affected a single lobe and there was no obvious metastasis to lymph nodes, hemithyroidectomy and central zone lymph node **dissection** were performed. Patients with ETE received total thyroidectomy. Patients who had lateral cervical lymph node involvement discovered by ultrasonography, **computed tomography**, or **magnetic resonance imaging** received modified radical neck dissection. Generally, neck dissection was not performed prophylactically.^[12,20–22,24]^ According to the data in our study, the patients with mETE had a **worse** prognosis including a lower DFS rate and an increased recurrence rate compared with the patients without ETE, perhaps because the mETE group had larger tumor sizes, higher rate of bilateral involvement, and more frequent lymph node metastasis than the group without ETE.

The important implications deriving from our research make it necessary to further improve pathology practice in surgery and to reach consensus on the exact meaning of mETE.

In general, pathological examination of lymph nodes in operation is performed when they are of larger size or suspected of metastasis. However, the result of our study revealed the importance of early diagnosis of mETE and emphasized the necessity of the examination of the entire specimen at surgery. If we are to include mETE in the pathological result, the consensus on the meaning of mETE should be reached. Histopathologically demonstrated extension to the sternothyroid muscle, thyroid capsule, or perithyroid soft tissue is defined as true mETE in the PTC setting. It should be separated from maximal ETE and different therapeutic strategy should be taken among PTC patients. Since PTC is the most frequently seen thyroid malignancy, it is suggested that the prognostic effect of mETE should be evaluated in PTC.

The result of this study should be viewed with its limitations. First of all, our systematic review yielded from 8 retrospective cohort studies. In a prospective cohort study, Hotomi et al^[21]^ discovered that PTC patients with ETE had a higher disease recurrence (RR = 1.97, 95% CI: 1.02–3.81, *P* = 0.04), but there was no significant difference in the DFS of PTC patients with mETE and no ETE (HR = 1.39, 95% CI: 0.76–2.55, *P* = 0.29). According to the Newcastle–Ottawa Scale score, the quality of some studies included was not much satisfactory, indicating the chance of possible bias. However, it seems that the qualities of study influence only recurrence and they have little impact on the connection between mETE and prognosis. Another limitation is due to the inconsistency in the definition of ETE. Besides, it is not easy to distinguish mETE, no ETE, as well as maximal ETE until surgery in some cases. Third, the data about the DFS were extracted from Kaplan–Meier curves to get HRs with 95% CIs with the above-mentioned method.^[15]^ Although we have checked many times, we could get the very similar results but not the original data. Although the methods we used have their limitations on getting the individual patient data from the studies included, they are the most acceptable method to analyze time-to-event outcomes when the individual patient data are inaccessible or the methods are not feasible. Apart from mETE, other probable factors may influence the prognosis of those with PTC, such as age at diagnosis, male gender, and tumor diameter.^[33–35]^

ETE diagnosed pathologically is related to lymph node metastasis, a positive margin, and vascular invasion.^[10]^ The significance of tumor margin status in patients with mETE should have been included in the study as it might influence the interpretation of the aggressiveness of mETE. However, there was only 1 article that mentioned the related data in which the result showed no significant difference between positive tumor margin and the prognosis of patients with mETE.^[12]^ Therefore, because of the limited original data, we could not analyze the impact of tumor margin status on patients with mETE, which should be further studied when there were sufficient data.

In conclusion, in this study, we indicated that mETE, similar to maximal ETE, was also a risk factor for poor prognosis in patients with PTC due to a higher rate of recurrence and decreased DFS. The impact of mETE on PTC clarified in our study contributes to the prognostic prediction in patients with PTC. Moreover, the role of mETE as a prognostic factor for PTC should be further investigated, so that it can be applied in the clinical practice.

## Supplementary Material

Supplemental Digital Content
