# Supplementary figures and images for: Prognostic impact of minimal extrathyroidal extension in papillary thyroid carcinoma
Source: Medicine (Baltimore). 2016 Dec 30;95(52):e5794. doi: 10.1097/MD.0000000000005794 (PMC5207600; doi:10.1097/MD.0000000000005794)

Supplyment Figure. Forest plots RD of recurrence in association with mETE in PTC


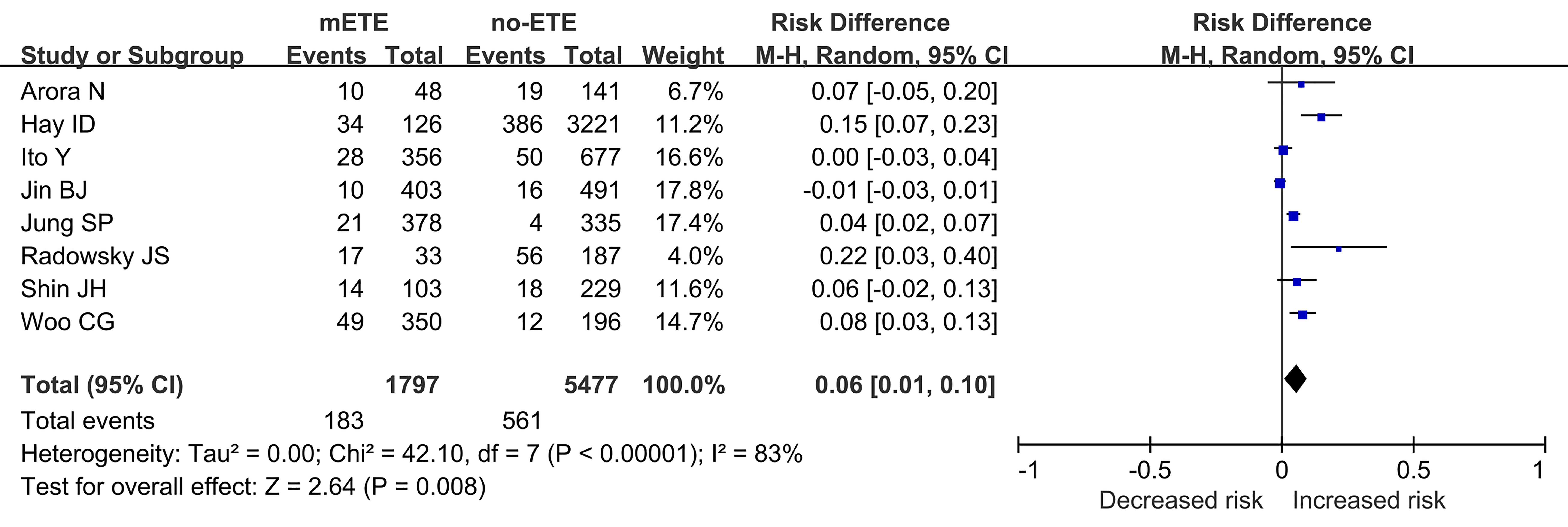

Supplement: Supplemental Digital Content [file medi-95-e5794-s001.doc]
